# Nomogram for predicting lymph node metastasis in ypT0–2 rectal cancer after neoadjuvant chemoradiotherapy

**DOI:** 10.1007/s00384-026-05201-y

**Published:** 2026-07-18

**Authors:** Hyeung-min Park, Jaram Lee, Soo Young Lee, Chang Hyun Kim, Hyeong Rok Kim

**Affiliations:** Department of Surgery, Chonnam National University Hwasun Hospital and Medical School, Hwasun, Republic of Korea

**Keywords:** Rectal cancer, Neoadjuvant chemoradiotherapy, Lymph node metastasis, Nomogram, Organ preservation

## Abstract

**Purpose:**

Accurate prediction of lymph node (LN) metastasis is essential for determining completion radical surgery following local excision (LE) in rectal cancer treated with neoadjuvant chemoradiotherapy (CRT). This study aimed to identify predictors of LN metastasis and develop a nomogram to support post-excision decision-making in rectal cancer patients after CRT.

**Methods:**

We retrospectively reviewed 215 patients with pathological stage ypT0–2 rectal cancer who underwent radical surgery following CRT. Pre- and post-CRT magnetic resonance imaging findings and clinicopathological factors were analysed to identify predictors of LN metastasis. A predictive nomogram was subsequently constructed and evaluated using receiver operating characteristic (ROC) curve, calibration curve, decision curve analysis (DCA), and clinical impact curve (CIC).

**Results:**

Factors associated with LN metastasis included pre-CRT mrN stage (mrN0 7.3% vs. mrN1 19.4% vs. mrN2 25.0%; *p* = 0.004), ypT stage (ypT0 2.7% vs. ypT1 13.3% vs. ypT2 19.8%; *p* = 0.003), and perineural invasion (PNI) (negative 11.1% vs. positive 35.3%; *p* = 0.013). Multivariable analysis identified pre-CRT mrN stage (*p* = 0.011), ypT stage (*p* = 0.033), and PNI (*p* = 0.036) as independent predictors. The nomogram exhibited acceptable calibration, with the area under the ROC curve of 0.777 (95% CI 0.681–0.874). DCA and CIC revealed the model’s potential clinical utility.

**Conclusion:**

Pre-CRT mrN stage, ypT stage, and PNI were independent predictors of LN metastasis in ypT0–2 rectal cancer following CRT. The developed nomogram may help identify candidates for completion radical surgery after LE, potentially facilitating organ preservation and reducing unnecessary surgical morbidity.

**Supplementary Information:**

The online version contains supplementary material available at https://doi.org/10.1007/s00384-026-05201-y.

## Introduction

For decades, locally advanced rectal cancer was treated with preoperative chemoradiotherapy (CRT) followed by radical surgery [1], as this approach promoted tumour downstaging, improved sphincter preservation, and reduced local recurrence [2, 3]. While total neoadjuvant therapy (TNT) has become the standard neoadjuvant treatment [4, 5], neoadjuvant CRT is still implemented based on national insurance policies and patient-specific indications.

Perioperative complications have been reported in approximately 30% of patients undergoing radical surgery for rectal cancer, including low anterior resection syndrome, urinary dysfunction, and sexual dysfunction [6–9]. Additionally, 10–17% of temporary stomas created during radical surgery fail to be reversed [10–12]. Given these challenges, organ-preserving approaches, such as local excision (LE), are increasingly considered for patients exhibiting a favourable tumour response to neoadjuvant therapy [13, 14]. In cases where post-CRT tumour remnants are confined to the muscularis propria, LE offer a viable alternative to radical surgery. However, even when LE achieves negative surgical margins, the risk of residual lymph node (LN) metastasis remains, necessitating meticulous patient selection [15].

Several studies have investigated predictors of LN metastasis in patients with ypT0–2 rectal cancer who underwent radical surgery following CRT [16–18]. These studies were based on the premise that residual LN metastasis after LE could contribute to recurrence; therefore, predictors of LN metastasis in patients undergoing radical surgery may also predict recurrence after LE [19, 20]. However, comprehensive studies incorporating both pre- and post-CRT magnetic resonance imaging (MRI) assessments remain limited. This study aimed to evaluate clinicopathological factors along with pre- and post-CRT MRI findings and to develop a predictive nomogram for LN metastasis risk. The nomogram is intended to help clinicians determine whether LE alone provides adequate local control or whether completion radical surgery is required to ensure oncological safety in patients with ypT0–2 rectal cancer after CRT.

## Methods

This study received approval from the institutional review board (IRB No. CNUHH-2024–244); informed consent was waived because of the retrospective design. This cohort study followed the STROBE (Strengthening the Reporting of Observational Studies in Epidemiology) reporting guidelines [21]. We conducted a retrospective analysis of patients with rectal cancer who underwent CRT followed by radical surgery at Chonnam National University Hwasun Hospital, Republic of Korea, between April 2013 and May 2020. All included patients underwent pre- and post-CRT pelvic MRI for clinical staging and had a confirmed pathological stage of ypT0–2. The enrolled patients included those diagnosed with locally advanced mid-to-low rectal cancer (cT3–4 N0 or cT any, N +) based on pre-CRT MRI. The cohort also included patients with low rectal cancer who received neoadjuvant CRT for anal sphincter preservation. Patients were excluded if they had distant metastasis at diagnosis, underwent emergency surgery, had non-adenocarcinoma histology, presented with synchronous or recurrent cancer, had incomplete clinicopathological data, or received TNT. Low and mid rectal cancers were defined as tumours with distal margins located < 5 cm and 5–10 cm from the anal verge, respectively, as determined by pre-CRT MRI.

Neoadjuvant CRT was initiated 2–4 weeks after baseline MRI. The CRT regimen consisted of pelvic radiotherapy with a total dose of 48.6–50.4 Gy delivered in 25–28 fractions [22]. Concurrent oral capecitabine (1650 mg/m^2^/day) was administered only on radiotherapy days. Restaging MRI was performed 4–6 weeks after CRT completion.

All MRI examinations were performed using a 3.0-Tesla system (Trio Tim, Siemens Healthcare) equipped with pelvic phased-array surface coils. To minimize motion artefacts from bowel motility, patients received intravenous hyoscine butylbromide (20 mg); no endorectal air insufflation or bowel preparation was used. Image acquisition parameters were standardized as follows: slice thickness of 4 mm; repetition/echo times 2850–5720/84–122 ms; field of view of 180–270 × 180–225 mm^2^; matrix size of 256–384 × 256–320. Two gastrointestinal radiologists, each with more than 5 years of subspecialty experience, independently interpreted the scans while blinded to clinical outcomes and pathology reports. Nodal involvement was assessed on T2-weighted and diffusion-weighted sequences using predefined morphological criteria: short-axis diameter 5 mm or more, irregular margins, and signal heterogeneity [23]. Clinical nodal stage was defined by the number of suspicious nodes: mrN1 for 1–3 nodes and mrN2 for 4 or more.

Radical surgery, consisting of total mesorectal excision, was performed 8–10 weeks following CRT in accordance with oncological surgical principles [24–26]. Immediately after specimen extraction, histopathological examination was performed, and an experienced pathology assistant ensured accurate retrieval of LNs. Tumour size was defined as the longest lesion diameter in the surgical specimen. Pathological staging was determined according to the 8th edition of the American Joint Committee on Cancer Staging Manual. Additionally, the presence of perineural invasion (PNI) and lymphovascular invasion (LVI) was assessed; PNI was defined as tumor cells within or surrounding the nerve sheath, while LVI was defined by the presence of tumor cells within the endothelium-lined channels. The tumor regression grade (TRG) was categorized as follows [27]: grade 1 (minor regression), ≤ 25% fibrosis within the tumor mass; grade 2 (moderate regression), 26%–50% fibrosis; grade 3 (major regression), > 50% tumor regression; and grade 4 (complete regression), presence of a fibrotic mass only without viable tumor cells.

Student's t-test or Mann–Whitney U test was used for continuous variables, and the chi-squared test or Fisher's exact test for categorical variables. Variables with *p* < 0.1 in univariate analyses were included in multivariable analyses. Predictive factors for LN metastasis were examined using backward stepwise multivariable logistic regression. *p* < 0.05 was considered statistically significant. A nomogram to predict LN metastasis was constructed based on the multivariable logistic model using the *rms* package. Model performance was assessed using receiver operating characteristic (ROC) curve analysis and calibration curves. To account for potential over-optimization, internal validation was performed with 1,000 bootstrap resamples to calculate the optimism-corrected area under the curve (AUC) and assess the agreement between predicted and observed probabilities. Clinical utility of the nomogram was assessed using decision curve analysis (DCA) to estimate net gains and a clinical impact curve (CIC) to examine its performance across various risk thresholds. Additionally, subgroup analyses were performed based on tumor location (low vs. mid rectal cancer) to evaluate the consistency of the predictive model, using the same univariate and multivariable regression approaches applied to the total cohort. Statistical analyses were performed using SPSS Statistics (version 27.0; IBM Corp., Armonk, NY, USA) and R (version 4.1.2).

## Results

**Fig. 1:**
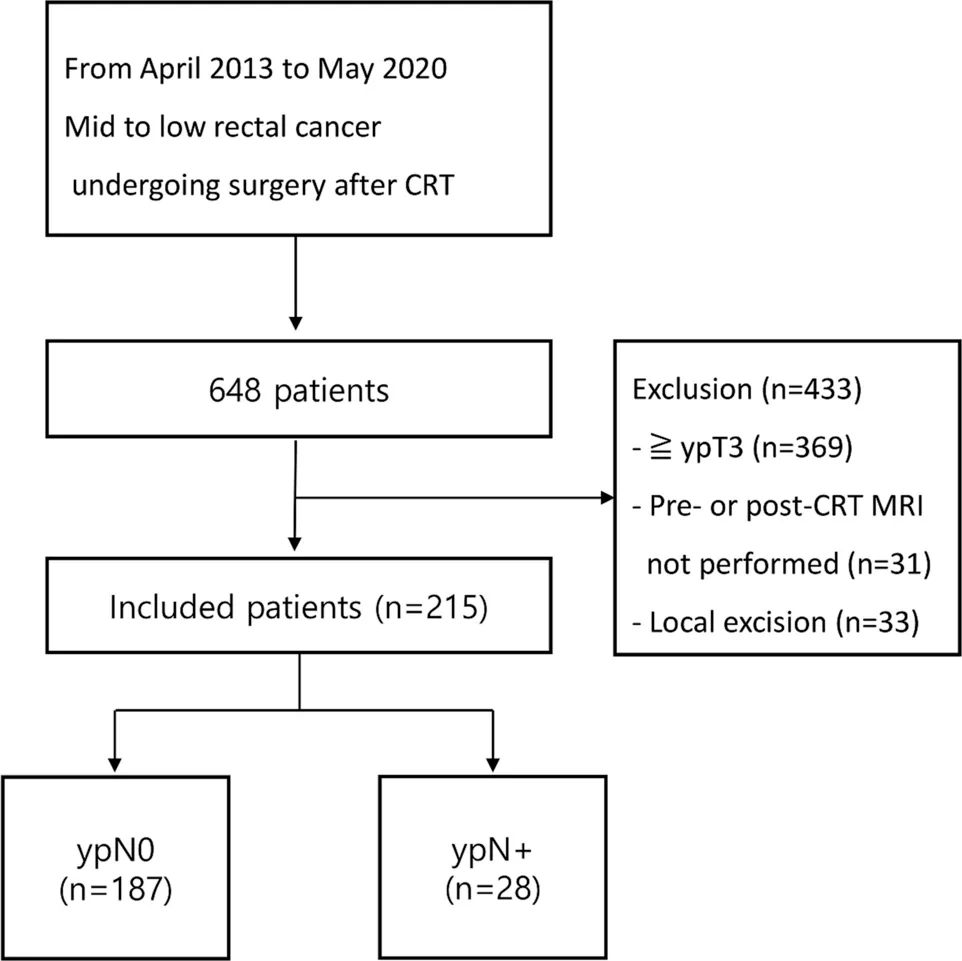
Flowchart of patient inclusion. Abbreviations: CRT, chemoradiotherapy; MRI, magnetic resonance imaging

A retrospective review identified 648 patients with mid-to-low rectal cancer who underwent surgery after CRT during the study period. After exclusions, 215 patients were included in the final cohort (Fig. 1). Pathological LN metastasis was confirmed in 28 (13.0%). Median age was 65 years (range 34–89). Overall, 149 (69.3%) were male, and 130 (60.4%) had low rectal cancer. ypT stage was ypT0 in 74 (34.4%), ypT1 in 30 (14.0%), and ypT2 in 111 (51.6%). The mean number of retrieved LNs was 28 (range 9–97), and the mean tumour size was 2.8 cm (range 0–18 cm).

Univariate analysis assessed associations between clinicopathological factors and LN metastasis (Table 1). The incidence of LN metastasis increased significantly with higher pre-CRT mrN stage (mrN0 7.3% vs. mrN1 19.4% vs. mrN2 25.0%; *p* = 0.004). Higher ypT stage was also associated with LN metastasis (ypT0 2.7% vs. ypT1 13.3% vs. ypT2 19.8%; *p* = 0.003), as was PNI (negative 11.1% vs. positive 35.3%; *p* = 0.013). Post-CRT ymrN stage showed a trend towards increased LN metastasis but did not reach statistical significance (ymrN0 11.9% vs. ymrN1 28.6%; *p* = 0.091).

**Table 1:** Clinicopathologic characteristics based on lymph node metastasis

Variables		Lymph node metastasis (-)	Lymph node metastasis (+)	*p*
Sex	Male	130 (87.2%)	19 (12.8%)	0.859
	Female	57 (86.4%)	9 (13.6%)	
Age (yr)	< 70	114 (89.1)	14 (10.9%)	0.270
	≥ 70	73 (83.9%)	14 (16.1%)	
BMI (kg/m^2^)	< 25	132 (88.6%)	17 (11.4%)	0.291
	≥ 25	55 (83.3%)	11 (16.7%)	
ASA score	1, 2	162 (88.0%)	22 (12.0%)	0.382
	3, 4	24 (82.8%)	5 (17.2%)	
Location	Middle	73 (85.9%)	12 (14.1%)	0.700
	Lower	114 (87.7%)	16 (12.3%)	
Pre-CRT	< 5	122 (88.4%)	16 (11.6%)	0.384
CEA (ng/mL)	≥ 5	64 (84.2%)	12 (15.8%)	
Pre-CRT	2	28 (90.3%)	3 (9.7%)	0.826
mrT stage	3	145 (86.3%)	23 (13.7%)	
	4	14 (87.5%)	2 (12.5%)	
Pre-CRT	0	115 (92.7%)	9 (7.3%)	0.004
mrN stage	1	54 (80.6%)	13 (19.4%)	
	2	18 (75.0%)	6 (25.0%)	
Post-CRT	0	87 (90.6%)	9 (9.4%)	0.104
ymrT stage	1	13 (86.7%)	2 (13.3%)	
	2	32 (88.9%)	4 (11.1%)	
	3	50 (80.6%)	12 (19.4%)	
	4	5 (83.3%)	1 (16.7%)	
Post-CRT	0	177 (88.1%)	24 (11.9%)	0.091
ymrN stage	1	10 (71.4%)	4 (28.6%)	
ypT stage	0	72 (97.3%)	2 (2.7%)	0.003
	1	26 (86.7%)	4 (13.3%)	
	2	89 (80.2%)	22 (19.8%)	
Tumor size (cm)	< 2.5	89 (87.3%)	13 (12.7%)	0.908
	≥ 2.5	98 (86.7%)	15 (13.3%)	
Lymphovascular	Negative	183 (87.1%)	27 (12.9%)	0.506
invasion	Positive	4 (80.0%)	1 (20.0%)	
Perineural	Negative	176 (88.9%)	22 (11.1%)	0.013
invasion	Positive	11 (64.7%)	6 (35.3%)	
TRG	1, 2	15 (83.3%)	3 (16.7%)	0.712
	3, 4	171 (87.2%)	25 (12.8%)	
Differentiation	w/d	82 (90.1%)	9 (9.9%)	0.170
	m/d	104 (85.2%)	18 (14.8%)	
	p/d	1 (50.0%)	1 (50.0%)	

*BMI*, body mass index; *ASA*, American Society of Anesthesiologists; *CRT*, chemoradiotherapy; *CEA*, carcinoembryonic antigen; *TRG*, tumor regression grade; *w/d*, well-differentiated; *m/d*, moderately differentiated; *p/d*, poorly differentiated

Multivariable analysis identified independent predictors of LN metastasis (Table 2). Pre-CRT mrN stage remained independently associated with LN metastasis (*p* = 0.011), with higher odds in mrN1 (odds ratio [OR] 3.064, 95% confidence interval [CI] 1.168–8.038) and mrN2 (OR 5.828, 95% CI 1.671–20.326) compared to mrN0. ypT stage was also independently associated (*p* = 0.033), with higher odds in ypT1 (OR 5.412, 95% CI 0.892–32.820) and ypT2 (OR 7.599, 95% CI 1.653–34.931) relative to ypT0. PNI was an independent predictor of LN metastasis (OR 3.636, 95% CI 1.084–12.187; *p* = 0.036).

**Table 2:** Multivariable analysis of predictive factors for lymph node metastasis

Variables		Odds ratio (95% confidence interval)	*p*
Pre-CRT mrN stage	0	Reference	0.011
	1	3.064 (1.168–8.038)	
	2	5.828 (1.671–20.326)	
ypT stage	0	Reference	0.033
	1	5.412 (0.892–32.820)	
	2	7.599 (1.653–34.931)	
Perineural invasion	Negative	Reference	0.036
	Positive	3.636 (1.084–12.187)	

Backward logistic regression analysis, step 2

Variables entered into step 1 were pre-CRT mrN stage, post-CRT ymrN stage, ypT stage, and perineural invasion

*CRT*, chemoradiotherapy

**Fig. 2:**
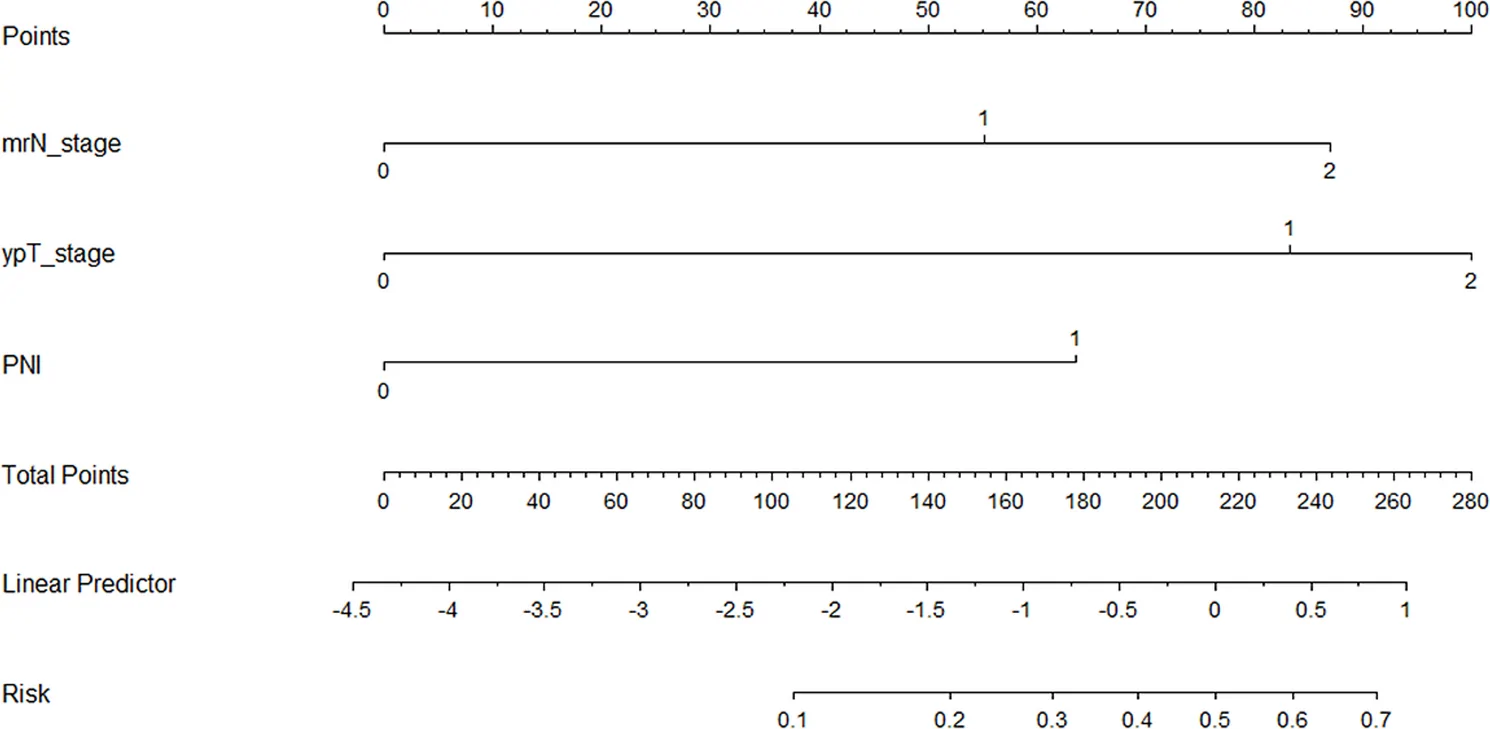
Nomogram for predicting the risk of lymph node metastasis. Points are assigned based on pre-CRT mrN stage, ypT stage, and perineural invasion (PNI) status; total points indicate the predicted probability of lymph node involvement. Abbreviations: CRT, chemoradiotherapy; PNI, perineural invasion

**Fig. 3:**
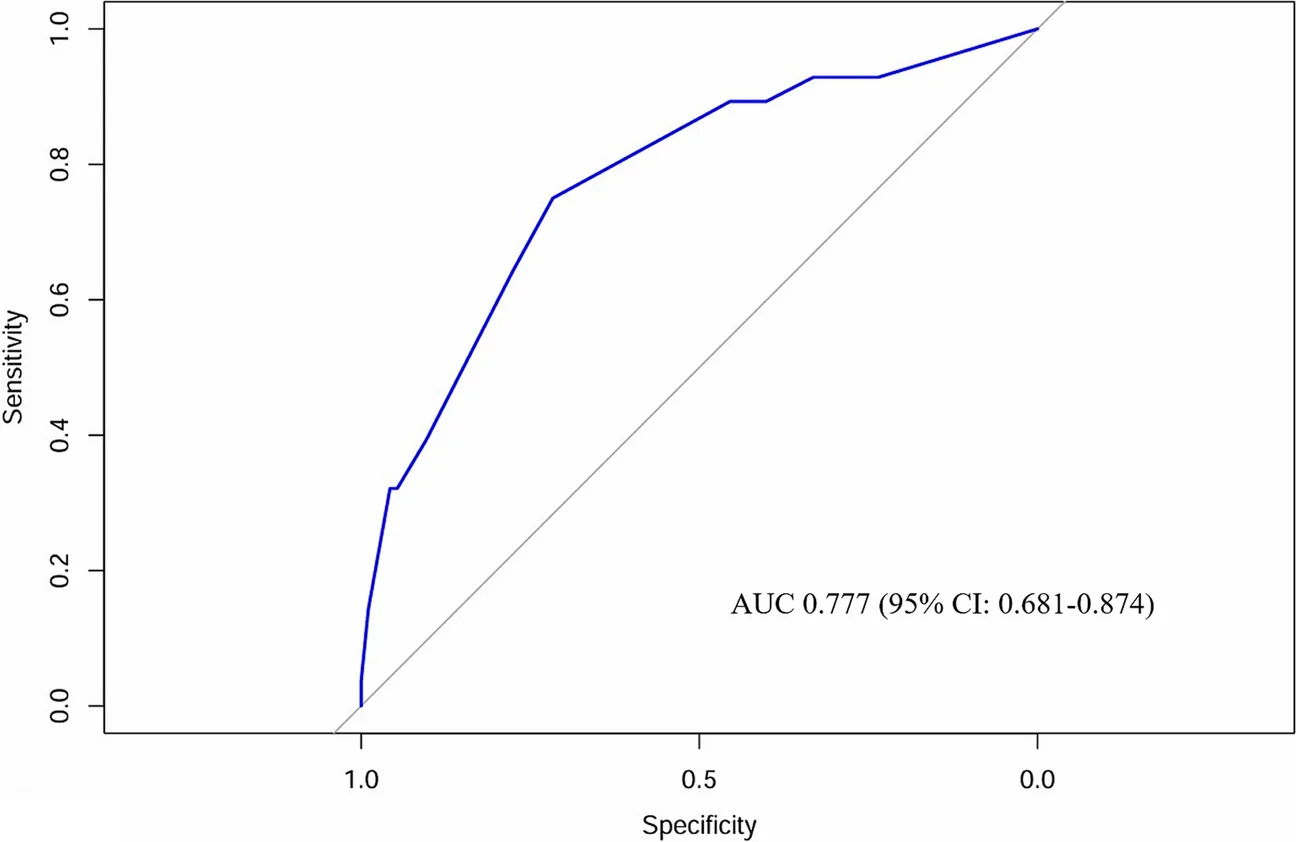
Receiver operating characteristic curve for the nomogram. The receiver operating characteristic curve (blue) yielded an area under the curve (AUC) of 0.777 (95% confidence interval: 0.681–0.874)

**Fig. 4:**
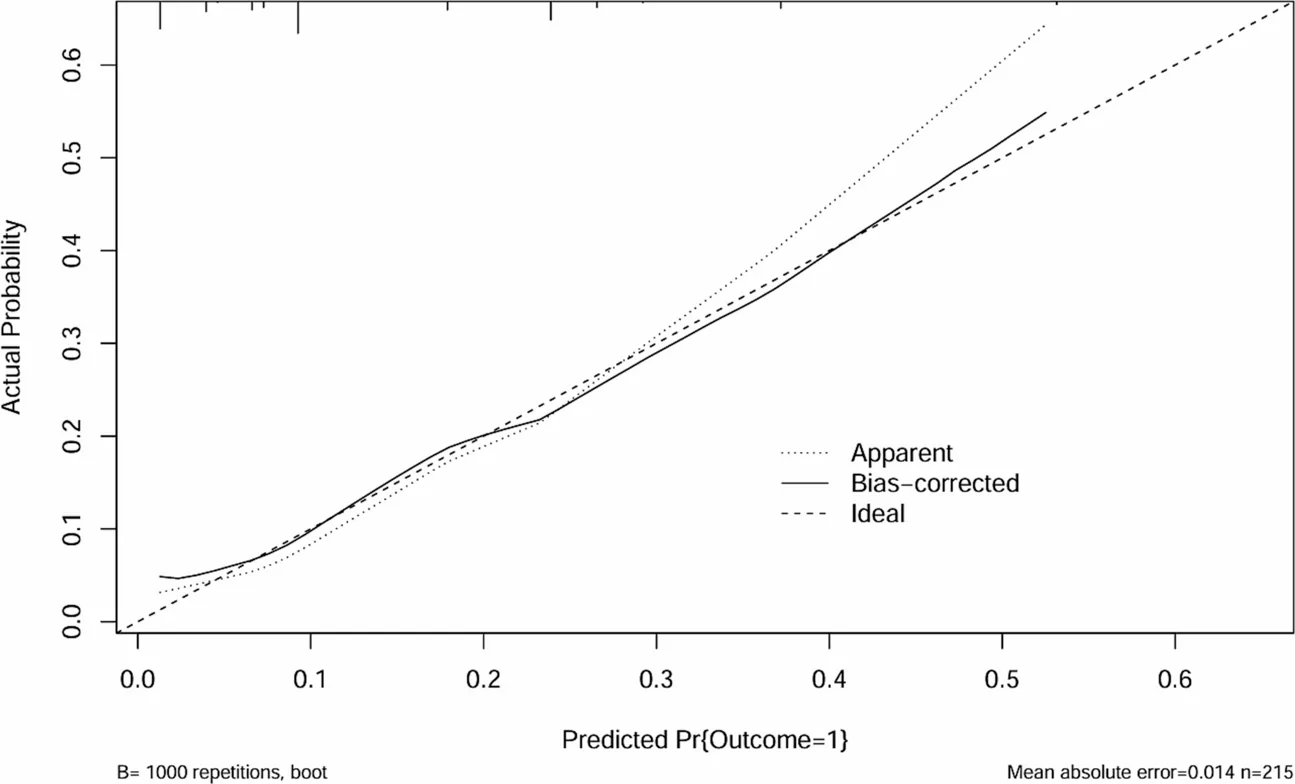
Calibration curve for the nomogram. The x-axis shows the predicted probability of lymph node metastasis, and the y-axis shows the observed probability. The dashed line indicates the ideal prediction, while the dotted and solid lines represent the apparent and bias-corrected performance of the nomogram, respectively. The bias-corrected curve was generated through internal validation with 1,000 bootstrap resamples (optimism-corrected AUC = 0.750). The plot shows a mean absolute error of 0.014

**Fig. 5:**
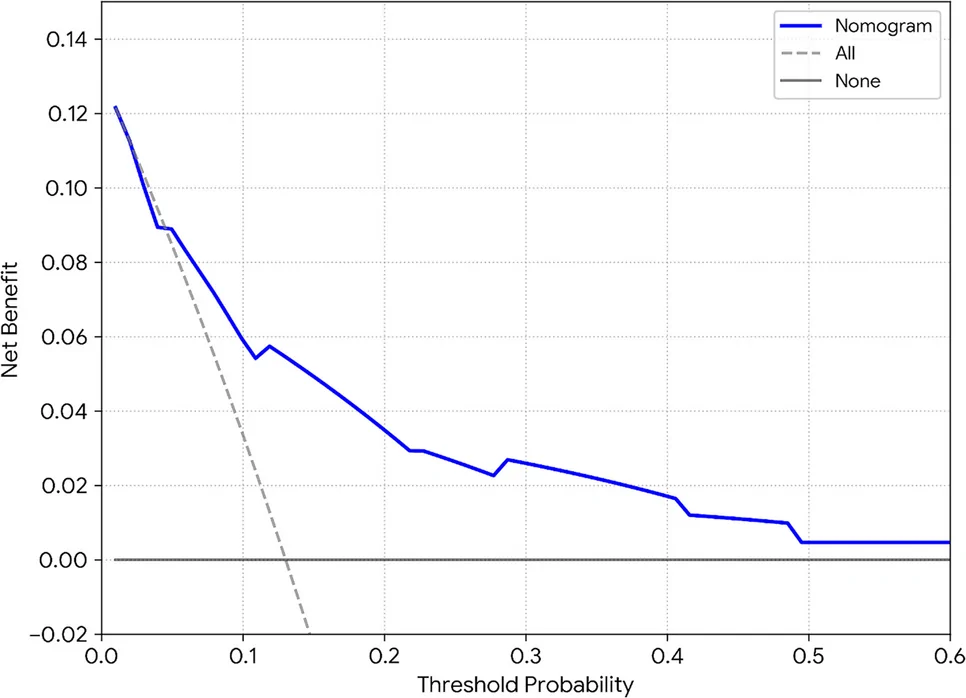
Decision curve analysis. The x-axis shows the threshold probability, and the y-axis shows the net benefit. The solid blue line (Nomogram) indicates net benefit compared to the ‘treat-all’ (grey dashed line) and ‘treat-none’ (horizontal black line) strategies

**Fig. 6:**
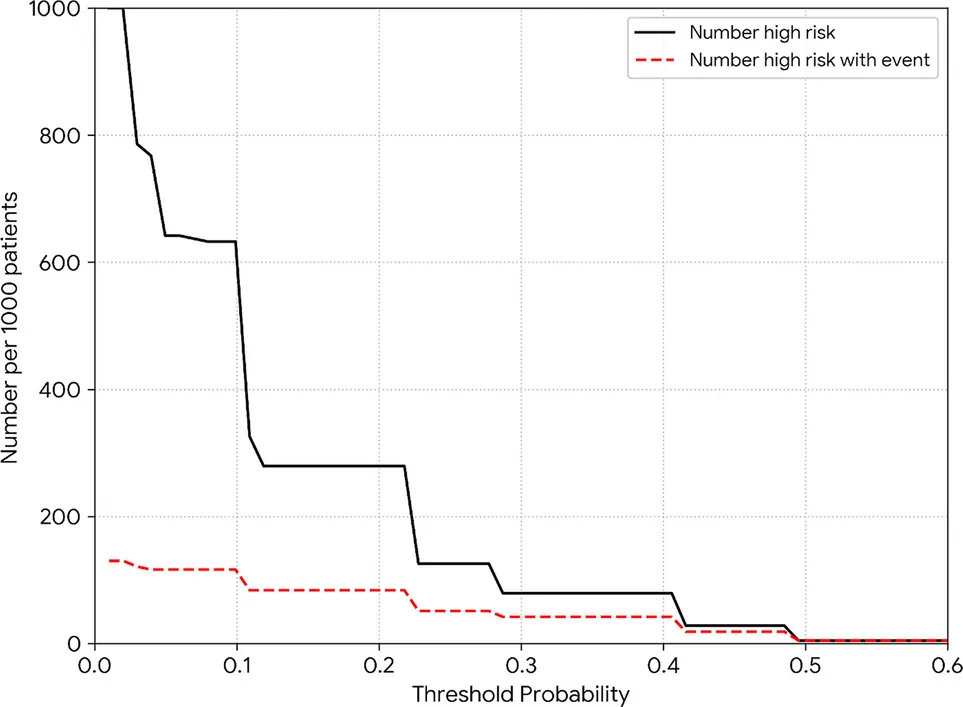
Clinical impact curve. The x-axis shows the threshold probability, and the y-axis shows the number of patients classified as high-risk per 1000 individuals. The solid black line indicates the number of patients classified as high-risk by the nomogram, while the red dashed line represents the actual number of patients with lymph node metastasis

Based on these predictors, a nomogram was developed to predict the risk of LN metastasis (Fig. 2). The nomogram yielded an apparent AUC of 0.777 (95% CI: 0.681–0.874; Fig. 3). After internal validation using 1,000 bootstrap resamples to account for model optimism, the optimism-corrected AUC was 0.750, indicating reliable predictive power. The calibration curve showed good agreement between predicted and observed probabilities (mean absolute error 0.014; Fig. 4). DCA revealed a potential net benefit compared with the 'treat-all' and 'treat-none' strategies across threshold probabilities of 5%–45% (Fig. 5). The CIC exhibited the concordance between predicted high-risk individuals and observed metastasis cases, indicating the model’s potential utility in guiding clinical decisions across various risk thresholds (Fig. 6).

To evaluate the consistency of the predictive model across different anatomical locations, we performed subgroup analyses for low and mid rectal cancer. In the low rectal cancer subgroup (n = 130), which is the most relevant group for organ preservation, multivariable analysis confirmed that pre-CRT mrN stage (*p* < 0.001), ypT stage (*p* = 0.036), and PNI (*p* = 0.025) were independent predictors of LN metastasis (Supplementary Tables 1 and 2). In the mid rectal cancer subgroup (n = 85), ypT stage (*p* = 0.026) and body mass index (BMI) (*p* = 0.003) were identified as independent predictors (Supplementary Tables 3 and 4).

## Discussion

We analysed predictors for LN metastasis in rectal cancer patients with ypT0–2 following CRT, incorporating both pre- and post-CRT MRI assessments. Pre-CRT mrN stage, ypT stage, and PNI were independent predictors of LN metastasis. The nomogram developed based on these variables exhibited good predictive performance. DCA and CIC supported the nomogram’s potential clinical utility.

Several studies have investigated clinicopathological factors associated with LN metastasis in ypT0–2 rectal cancer after CRT. One study of patients who underwent MRI before CRT identified MRI-defined mesorectal fascia involvement, high-grade histopathology, advanced ypT stage, and PNI as significant predictors [16]. However, 22.3% of the cohort received TNT with induction or consolidation chemotherapy, and the median number of retrieved LNs was 8 below the recommended threshold of 12 [28]. Von Den Grün et al. [17] analysed 776 ypT0–2 patients from two randomised controlled trials and found that advanced ypT stage, lymphatic invasion, and residual tumour diameter ≥ 10 mm independently predicted LN metastasis. In that study, clinical staging before CRT used MRI, endorectal ultrasound, and CT, which may have introduced diagnostic heterogeneity. Bosch et al. [18] reported associations between LN metastasis and clinical nodal involvement, high-grade histopathology, and residual tumour diameter ≥ 10 mm. However, the median number of examined LNs was only 7, which may have compromised the validity of their findings.

MRI has been widely explored as a tool for predicting LN metastasis in rectal cancer [29–32]. A study analysing patients from the OPRA trial suggested that baseline MRI could help predict the feasibility of organ preservation [33, 34]. In contrast, post-CRT MRI has been reported to have low accuracy for evaluating residual LN metastasis [23, 35, 36]. This limitation has been attributed to radiation-induced changes in the rectal wall and mesorectal nodes that compromise imaging assessment [37, 38]. Consistent with these reports, pre-CRT mrN stage independently predicted LN metastasis in our study, whereas post-CRT MRI findings were not significantly associated with LN metastasis.

Numerous studies have linked ypT stage with the risk of LN metastasis in rectal cancer patients who underwent surgery following CRT [16–19, 39]. Reported rates of residual LN metastasis increased with higher ypT stages but varied across studies because of differences in design, inclusion criteria, patient characteristics, and cohort sizes (ypT0, 2.2–17.4%; ypT1, 2.3–20.8%; ypT2, 16.9–25.8%). Similarly, ypT stage was a significant predictor in our model, with increasing risk at more advanced stages (ypT0 2.7% vs. ypT1 13.3% vs. ypT2 19.8%; *p* = 0.003).

PNI has been proposed as a predictor of LN metastasis and has also been associated with an increased risk of local recurrence [16, 40, 41]. In previous studies analysing ypT0–2 rectal cancer after CRT [16, 17], the risk of LN metastasis ranged from 11.3% to 16.3% without PNI, compared with 21.4% to 42.9% when PNI was present. Similarly, PNI was a significant predictor of LN metastasis in our study (negative 11.1% vs. positive 35.3%; *p* = 0.013). Because PNI can be assessed pathologically in the LE specimen, it may help determine the feasibility of organ preservation.

The favourable predictive performance and practical applicability of our nomogram supported its clinical utility. The nomogram achieved acceptable discriminative performance, with an AUC of 0.777 (95% CI: 0.681–0.874). In the calibration plot, a mean absolute error of 0.014 reflected minimal deviation between predicted and actual outcomes, supporting the model’s reliable calibration performance. To assess its clinical implications, we employed DCA and CIC, which are increasingly recognized as essential tools for evaluating the net clinical gain of a predictive model beyond mere statistical accuracy. DCA revealed higher net benefit for nomogram-guided decisions than 'treat-all' or 'treat-none' strategies across threshold probabilities of 5%–45%. CIC further showed accurate identification of high-risk individuals who would benefit from intervention, as evidenced by the curves aligning closely at higher thresholds. These findings suggested that our nomogram may guide clinicians in selecting suitable candidates for organ preservation among rectal cancer patients who underwent LE following CRT, potentially reducing unnecessary radical surgery. While our DCA indicated a potential net benefit within the 5%–45% threshold range, the selection of a specific threshold should be tailored to the clinical context. For instance, a lower threshold (e.g., 5%–10%) might be prioritized for younger, fit patients to maximize oncological safety, whereas a higher threshold (e.g., 15%–20%) could be considered for elderly patients or those with significant comorbidities to avoid surgical morbidity. However, as DCA and CIC are primarily designed to evaluate clinical utility relative to patient preferences or to compare different models, these results represent exploratory insights into clinical trade-offs rather than definitive clinical recommendations.

Research on organ preservation strategies after CRT in rectal cancer has been actively pursued [13, 14]. Recently, efforts have expanded to incorporate TNT, followed by a watch-and-wait approach or LE [42–45]. In the TNT era, LE serves as a diagnostic and therapeutic approach that confirms pathological outcomes while offering organ preservation for patients at low risk of LN metastasis. In our study, LE-derived data enabled evidence-based decisions on whether to continue organ preservation or proceed with completion radical surgery. As TNT becomes standard neoadjuvant treatment for locally advanced rectal cancer, identifying predictive factors for LN metastasis in ypT0–2 patients undergoing radical surgery after TNT may inform optimal organ-preserving strategies. Because patients who received TNT were excluded from this study, the present nomogram should not be directly extrapolated to patients treated with TNT. TNT may alter tumor regression, nodal sterilization, and the relationship between ypT stage and residual nodal metastasis. Therefore, TNT-specific predictive models should be developed and externally validated in future studies of ypT0–2 rectal cancer patients treated with modern neoadjuvant strategies.

Our subgroup analyses based on the anatomical level may provide further evidence regarding the potential clinical utility of the developed nomogram. In the low rectal cancer subgroup, the independent predictors were identical to those identified in the overall population, reinforcing the model's reliability in guiding post-excision decisions where organ preservation is most frequently considered. While the mid rectal cancer subgroup showed slight variation, such as the emergence of BMI as a predictor, the ypT stage remained a robust indicator of LN metastasis across both anatomical locations. Such consistency tentatively suggests that the core elements of the nomogram may maintain their clinical relevance for estimating lymph node metastasis risk across the mid-to-low rectum.

This study had several limitations. First, the retrospective design may have introduced selection bias. Second, the sample size was relatively small because of its single-centre setting, and the number of node-positive cases was particularly limited. Although the overall cohort included 215 patients, pathological LN metastasis was confirmed in only 28 individuals (13.0%). This limited number of events may not adequately represent the clinical and pathological heterogeneity of node-positive disease and could increase the risk of model instability. Third, although internal validation was performed using 1,000 bootstrap resamples, the absence of external validation using an independent cohort remains a primary limitation that may restrict the generalizability of our findings. Furthermore, since the study was conducted at a single Korean tertiary centre and included only mid-to-low rectal cancer patients, potential selection bias and specific institutional practice patterns may limit the applicability of the model to other populations, diverse ethnic groups, and other anatomical subgroups, such as upper rectal cancer. Therefore, future multicenter studies with external validation cohorts are essential to rigorously evaluate and calibrate this model across various clinical settings and ethnicities. Fourth, given the limited sample size and the historical nature of the cohort, several potentially relevant MRI and pathological variables could not be evaluated. During the study period, mesorectal fascia involvement and extramural vascular invasion were not consistently documented in structured MRI reports. Similarly, the pathological evaluation of tumor budding lacked standardized reporting protocols during this time. The omission of these variables may have affected the discriminative performance and calibration of the developed nomogram. Fifth, formal inter-reader agreement statistics, such as Kappa coefficients, were not assessed for MRI interpretations in this retrospective study. This absence may be considered a potential deficiency regarding the reliability of our imaging evaluation. However, to mitigate this limitation, all MRI scans were independently evaluated by two experienced gastrointestinal radiologists with extensive subspecialty experience who were blinded to clinical and pathological outcomes, thereby supporting the clinical validity of the staging results used in our analysis. Nevertheless, as only patients with both pre- and post-CRT MRI findings were included, our analysis could offer an advantage in the comprehensive investigation of predictors. Additionally, since LN examination was performed immediately on fresh tissue after surgery, the number of retrieved LNs exceeded the recommended threshold of 12 despite the influence of CRT, enhancing the reliability of our pathological outcomes. Furthermore, beyond ROC and calibration curves, DCA and CIC revealed the potential clinical utility of our nomogram in facilitating individualized decision-making and exploring tailored surgical strategies.

## Conclusion

We integrated pre- and post-CRT MRI findings to identify predictors of suitability for LE after CRT in patients with rectal cancer. The resulting nomogram may serve as a decision-support tool for selecting suitable candidates for organ preservation for whom LE alone might be sufficient. However, given the lack of external validation, these findings should be considered preliminary, and the model’s clinical efficacy remains to be established through further studies involving independent cohorts.

## Supplementary Information

Below is the link to the electronic supplementary material.

ESM 1 (DOCX 28.4 KB)

ESM 2 (DOCX 23.1 KB)

ESM 3 (DOCX 28.1 KB)

ESM 4 (DOCX 22.7 KB)

## Data Availability

The data that support the findings of this study are available from the corresponding author upon reasonable request.
