# Supplementary material for: Nomogram for predicting lymph node metastasis in ypT0–2 rectal cancer after neoadjuvant chemoradiotherapy
Source: Int J Colorectal Dis. 2026 Jul 18;41(1):162. doi: 10.1007/s00384-026-05201-y (PMC13661684; doi:10.1007/s00384-026-05201-y)
Supplement: Supplementary file 1 [file 384_2026_5201_MOESM1_ESM.docx]

**Supplementary Table 1.** Clinicopathologic characteristics based on lymph node metastasis in lower rectal cancer (n = 130)

| Variables | | Lymph node metastasis (-) | Lymph node metastasis (+) | *p* |
| --- | --- | --- | --- | --- |
| Sex | Male | 74 (88.1%) | 10 (11.9%) | 1.000 |
|  | Female | 40 (87.0%) | 6 (13.0%) |  |
| Age (yr) | < 70 | 69 (88.5%) | 9 (11.5%) | 0.744 |
|  | ≥ 70 | 45 (86.5%) | 7 (13.5%) |  |
| BMI (kg/m^2^) | < 25 | 69 (86.3%) | 11 (13.8%) | 0.527 |
|  | ≥ 25 | 45 (90.0%) | 5 (10.0%) |  |
| ASA score | 1, 2 | 97 (89.0%) | 12 (11.0%) | 0.703 |
|  | 3, 4 | 17 (85.0%) | 3 (15.0%) |  |
| Pre-CRT | < 5 | 76 (91.6%) | 7 (8.4%) | 0.074 |
| CEA (ng/mL) | ≥ 5 | 38 (80.9%) | 9 (19.1%) |  |
| Pre-CRT | 2 | 22 (91.7%) | 2 (8.3%) | 0.237 |
| mrT stage | 3 | 87 (87.9%) | 12 (12.1%) |  |
|  | 4 | 5 (71.4%) | 2 (28.6%) |  |
| Pre-CRT | 0 | 79 (96.3%) | 3 (3.7%) | <0.001 |
| mrN stage | 1 | 26 (78.8%) | 7 (21.2%) |  |
|  | 2 | 9 (60.0%) | 6 (40.0%) |  |
| Post-CRT | 0 | 54 (90.0%) | 6 (10.0%) | 0.159 |
| ymrT stage | 1 | 9 (90.0%) | 1 (10.0%) |  |
|  | 2 | 25 (92.6%) | 2 (7.4%) |  |
|  | 3 | 26 (81.3%) | 6 (18.8%) |  |
|  | 4 | 0 (0.0%) | 1 (100.0%) |  |
| Post-CRT | 0 | 108 (89.3%) | 13 (10.7%) | 0.081 |
| ymrN stage | 1 | 6 (66.7%) | 3 (33.3%) |  |
| ypT stage | 0 | 46 (97.9%) | 1 (2.1%) | 0.018 |
|  | 1 | 11 (78.6%) | 3 (21.4%) |  |
|  | 2 | 57 (82.6%) | 12 (17.4%) |  |
| Tumor size (cm) | < 2.5 | 51 (91.1%) | 5 (8.9%) | 0.308 |
|  | ≥ 2.5 | 63 (85.1%) | 11 (14.9%) |  |
| Lymphovascular | Negative | 111 (88.1%) | 15 (11.9%) | 0.413 |
| invasion | Positive | 3 (75.0%) | 1 (25.0%) |  |
| Perineural | Negative | 107 (89.9%) | 12 (10.1%) | 0.030 |
| invasion | Positive | 7 (63.6%) | 4 (36.4%) |  |
| TRG | 1, 2 | 13 (86.7%) | 2 (13.3%) | 1.000 |
|  | 3, 4 | 101 (87.8%) | 14 (12.2%) |  |
| Differentiation | w/d | 55 (91.7%) | 5 (8.3%) | 0.091 |
|  | m/d | 59 (85.5%) | 10 (14.5%) |  |
|  | p/d | 0 (0.0%) | 1 (100.0%) |  |

*BMI*, body mass index; *ASA*, American Society of Anesthesiologists; *CRT*, chemoradiotherapy; *CEA*, carcinoembryonic antigen; *TRG*, tumor regression grade; *w/d*, well-differentiated; *m/d*, moderately differentiated; *p/d*, poorly differentiated.
