# Supplementary material for: Nomogram for predicting lymph node metastasis in ypT0–2 rectal cancer after neoadjuvant chemoradiotherapy
Source: Int J Colorectal Dis. 2026 Jul 18;41(1):162. doi: 10.1007/s00384-026-05201-y (PMC13661684; doi:10.1007/s00384-026-05201-y)
Supplement: Supplementary file 2 [file 384_2026_5201_MOESM2_ESM.docx]

**Supplementary Table 2.** Multivariable analysis of predictive factors for lymph node metastasis in lower rectal cancer

| Variables | | Odds ratio (95% confidence interval) | *p* |
| --- | --- | --- | --- |
| Pre-CRT mrN stage | 0 | Reference | <0.001 |
|  | 1 | 6.754 (1.426–31.980) |  |
|  | 2 | 53.010 (7.209–389.806) |  |
| ypT stage | 0 | Reference | 0.036 |
|  | 1 | 39.315 (2.397–644.724) |  |
|  | 2 | 12.596 (1.205–131.648) |  |
| Perineural invasion | Negative | Reference | 0.025 |
|  | Positive | 8.213 (1.299–51.909) |  |

Variables were pre-CRT CEA, pre-CRT mrN stage, post-CRT ymrN stage, ypT stage, perineural invasion, and differentiation

*CRT*, chemoradiotherapy; *CEA*, carcinoembryonic antigen
