# Supplementary material for: Nomogram for predicting lymph node metastasis in ypT0–2 rectal cancer after neoadjuvant chemoradiotherapy
Source: Int J Colorectal Dis. 2026 Jul 18;41(1):162. doi: 10.1007/s00384-026-05201-y (PMC13661684; doi:10.1007/s00384-026-05201-y)
Supplement: Supplementary file 3 [file 384_2026_5201_MOESM3_ESM.docx]

**Supplementary Table 3.** Clinicopathologic characteristics based on lymph node metastasis in middle rectal cancer (n = 85)

| Variables | | Lymph node metastasis (-) | Lymph node metastasis (+) | *p* |
| --- | --- | --- | --- | --- |
| Sex | Male | 56 (86.2%) | 9 (13.8%) | 1.000 |
|  | Female | 17 (85.0%) | 3 (15.0%) |  |
| Age (yr) | < 70 | 45 (90.0%) | 5 (10.0%) | 0.219 |
|  | ≥ 70 | 28 (80.0%) | 7 (20.0%) |  |
| BMI (kg/m^2^) | < 25 | 63 (91.3%) | 6 (8.7%) | 0.008 |
|  | ≥ 25 | 10 (62.5%) | 6 (37.5%) |  |
| ASA score | 1, 2 | 65 (86.7%) | 10 (13.3%) | 0.610 |
|  | 3, 4 | 7 (77.8%) | 2 (22.2%) |  |
| Pre-CRT | < 5 | 46 (83.6%) | 9 (16.4%) | 0.531 |
| CEA (ng/mL) | ≥ 5 | 26 (89.7%) | 3 (10.3%) |  |
| Pre-CRT | 2 | 6 (85.7%) | 1 (14.3%) | 0.359 |
| mrT stage | 3 | 58 (84.1%) | 11 (15.9%) |  |
|  | 4 | 9 (100.0%) | 0 (0.0%) |  |
| Pre-CRT | 0 | 36 (85.7%) | 6 (14.3%) | 0.536 |
| mrN stage | 1 | 28 (82.4%) | 6 (17.6%) |  |
|  | 2 | 9 (100.0%) | 0 (0.0%) |  |
| Post-CRT | 0 | 33 (91.7%) | 3 (8.3%) | 0.373 |
| ymrT stage | 1 | 4 (80.0%) | 1 (20.0%) |  |
|  | 2 | 7 (77.8%) | 2 (22.2%) |  |
|  | 3 | 24 (80.0%) | 6 (20.0%) |  |
|  | 4 | 5 (100.0%) | 0 (0.0%) |  |
| Post-CRT | 0 | 69 (86.3%) | 11 (13.8%) | 0.542 |
| ymrN stage | 1 | 4 (80.0%) | 1 (20.0%) |  |
| ypT stage | 0 | 26 (96.3%) | 1 (3.7%) | 0.016 |
|  | 1 | 15 (93.8%) | 1 (6.3%) |  |
|  | 2 | 32 (76.2%) | 10 (23.8%) |  |
| Tumor size (cm) | < 2.5 | 38 (82.6%) | 8 (17.4%) | 0.347 |
|  | ≥ 2.5 | 35 (89.7%) | 4 (10.3%) |  |
| Lymphovascular | Negative | 72 (85.7%) | 12 (14.3%) | 1.000 |
| invasion | Positive | 1 (100.0%) | 0 (0.0%) |  |
| Perineural | Negative | 69 (87.3%) | 10 (12.7%) | 0.199 |
| invasion | Positive | 4 (66.9%) | 2 (33.3%) |  |
| TRG | 1, 2 | 2 (66.7%) | 1 (33.3%) | 0.374 |
|  | 3, 4 | 70 (86.4%) | 11 (13.6%) |  |
| Differentiation | w/d | 27 (87.1%) | 4 (12.9%) | 0.885 |
|  | m/d | 45 (84.9%) | 8 (15.1%) |  |
|  | p/d | 1 (100.0%) | 0 (0.0%) |  |

*BMI*, body mass index; *ASA*, American Society of Anesthesiologists; *CRT*, chemoradiotherapy; *CEA*, carcinoembryonic antigen; *TRG*, tumor regression grade; *w/d*, well-differentiated; *m/d*, moderately differentiated; *p/d*, poorly differentiated.
