# Supplementary material for: Nomogram for predicting lymph node metastasis in ypT0–2 rectal cancer after neoadjuvant chemoradiotherapy
Source: Int J Colorectal Dis. 2026 Jul 18;41(1):162. doi: 10.1007/s00384-026-05201-y (PMC13661684; doi:10.1007/s00384-026-05201-y)
Supplement: Supplementary file 4 [file 384_2026_5201_MOESM4_ESM.docx]

**Supplementary Table 4.** Multivariable analysis of predictive factors for lymph node metastasis in middle rectal cancer

| Variables | | Odds ratio (95% confidence interval) | *p* |
| --- | --- | --- | --- |
| BMI (kg/m^2^) | < 25 | Reference | 0.003 |
|  | ≥ 25 | 28.700 (3.066–268.759) |  |
| ypT stage | 0 | Reference | 0.026 |
|  | 1 | 1.310 (0.065–26.468) |  |
|  | 2 | 31.159 (1.899–511.281) |  |

Variables were BMI and ypT stage

*BMI*, body mass index
